# Evaluation of tumour motion and internal/external correlation in lung SABR

**DOI:** 10.1259/bjr.20220461

**Published:** 2023-07-10

**Authors:** Asmaa M Ali, Jason B Greenwood, Gerard M Walls, Louise Belshaw, Christina E Agnew, Jonathan McAleese, Glenn Whitten, Denise M Irvine, Alan R Hounsell, Conor K McGarry

**Affiliations:** 1 School of Mathematics and Physics, Queen’s University Belfast, Belfast, Northern Ireland; 2 Cancer Centre Belfast City Hospital, Belfast Health & Social Care Trust, Belfast, Northern Ireland; 3 Patrick G Johnston Centre for Cancer Research, Queen’s University Belfast, Lisburn Road, Belfast, Northern Ireland

## Abstract

**Objective:**

This study aims to analyse lung tumour motion and to investigate the correlation between the internal tumour motion acquired from four-dimensional computed tomography (4DCT) and the motion of an external surrogate.

**Methods:**

A data set of 363 4DCT images was analysed. Tumours were classified based on their anatomical lobes. The recorded gross tumour volume (GTV) information included the centroid GTV motion in the superior–inferior, anteroposterior and left-right directions, and in three-dimensions (3D). For the internal/external correlation, the RPM surrogate breathing signals of 260 patients were analysed via an in-house script. The external motion was correlated with the 3D centroid motion, and the maximum tumour motion via Spearman’s correlation. The effect of tumour volume on the amount of motion was evaluated.

**Results:**

The greatest 3D tumour amplitude was found for tumours located in the lower part of the lung, with a maximum of 26.7 mm. The Spearman’s correlation of the internal 3D motion was weak in the upper (*r* = 0.21) and moderate in the middle (*r* = 0.51) and the lower (*r* = 0.52) lobes. There was no obvious difference in the correlation coefficients between the maximum tumour displacement and the centroid motion. No correlation was found between the tumour volume and the magnitude of motion.

**Conclusion:**

Our results suggest that tumour location can be a good predictor of its motion. However, tumour size is a poor predictor of the motion.

**Advances in knowledge:**

This knowledge of the distribution of tumour motion throughout the thoracic regions will be valuable to research groups investigating the refinement of motion management strategies.

## Introduction

The accuracy of radiation dose delivery is compromised by organ motion for tumours in the thoracic cavity. To accommodate tumour motion, wide treatment margins are required. However, increased treatment toxicity is associated with widened margins. Numerous motion management techniques aimed at minimising the extent of the motion,^1^ such as breath-hold (BH) techniques, have been adopted. Furthermore, the motion-encompassing approach can be performed by acquiring slow CT and inhalation/exhalation BH CT. The gating technique is another way of managing respiratory motion and refers to the process of tracking the respiratory cycle in order to deliver the radiation dose to a certain portion. Gating the radiation beam can be achieved by tracking fiducial markers (FMs) as internal surrogates,^2^ or based on the use of external surrogates.^3^ Finally, four-dimensional CT (4DCT) images can be acquired to account for respiratory motion.^4^

4DCT has the potential to account for respiratory motion during the entire treatment process, starting from the image acquisition to the treatment delivery. It allows the patient’s internal organ motion to be quantified as a function of the respiratory cycle by acquiring oversampled data for each acquired slice,^5^ generating a spatiotemporal data set of the patient’s anatomical structures.^6^ Stereotactic ablative radiotherapy (SABR) is commonly used for early-stage non-small cell lung cancer (NSCLC),^7,8^ with targets of a maximum diameter less than 5 cm.^9,10^ Larger maximum target diameters have also been reported,^11^ however, there are insufficient data on the safety and efficacy for SABR in this setting at present.^10^ SABR has also been used for intra- and extracranial metastases to achieve long-term local control.^12,13^ SABR treatment for early stage NSCLC has been shown to offer superior treatment outcomes with high local control rates and manageable toxicities^14–16^ both as single- and multifraction regimens^17^ compared with conventional radiotherapy.^14^

**Figure 1.**
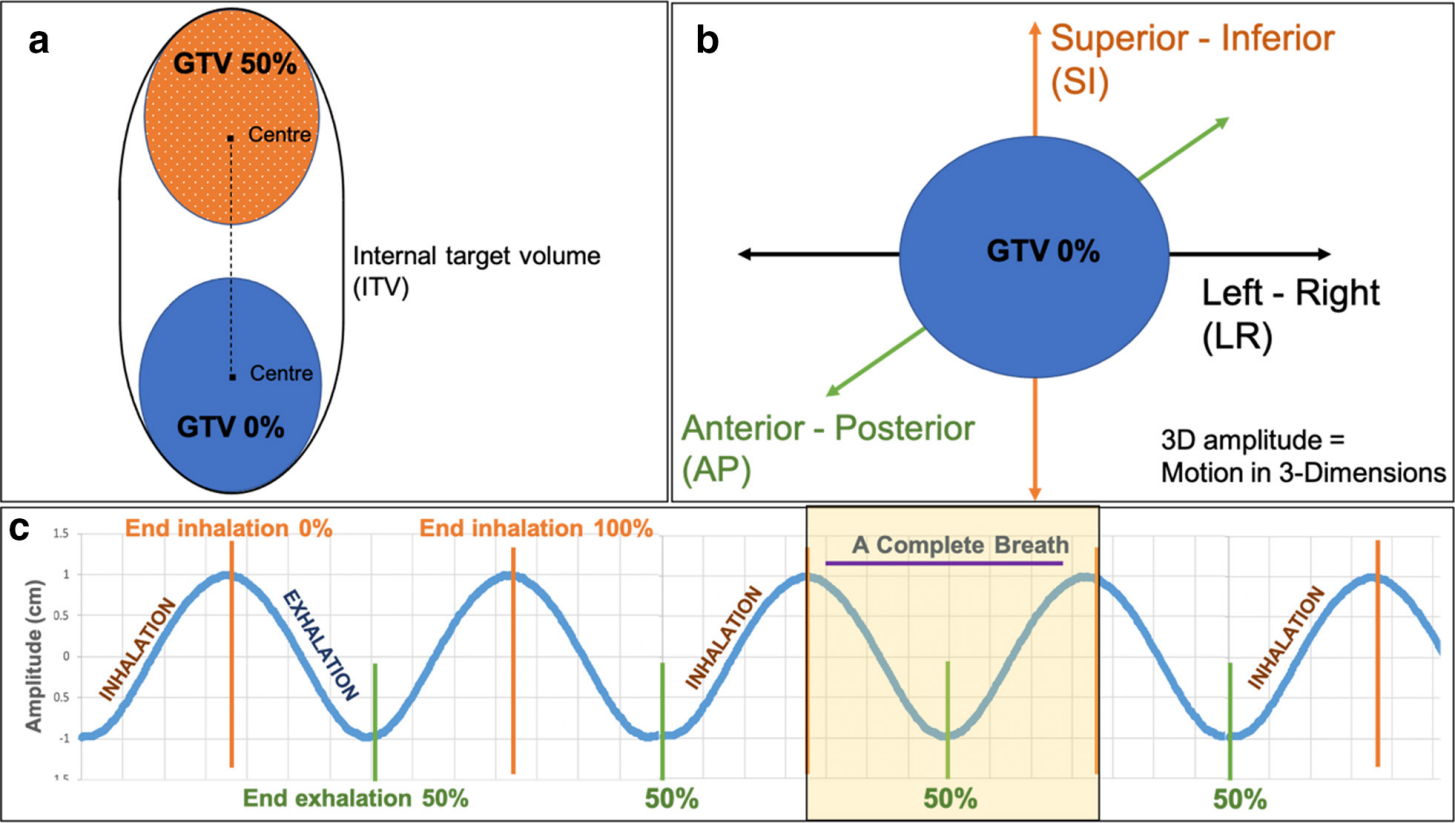
(**a**) An illustration of the centre of mass motion between the two breathing extremes, GTV0%, and GTV50%. Panel **b** shows tumour motion in SI, AP and LR directions. Panel c illustrates a breathing waveform with endinhalation (phase 0%), end-exhalation (phase 50%), and a complete breathing cycle. AP, anteroposterior; GTV, gross tumour volume; LR, left-right; SI, superior-inferior.

In comparison with conventional radiotherapy, in SABR, the therapeutic dose is limited to the gross tumour volume (GTV) with reduced margins, in order to minimise the volume of tissues exposed to the higher radiation dose. The clinical target volume (CTV), a margin introduced to account for the microscopic expansion of the disease in conventional treatments, is not typically used in SABR.^18,19^ However, for lung SABR, the internal target volume (ITV) is created to account for the movement of the GTV during breathing (Figure 1).^8^

One of the most widely adopted approaches for taking respiratory motion into account for thoracic SABR treatments is 4DCT,^8,9,12,20^ in which an external marker is also used to monitor patient breathing during the image acquisition for the radiotherapy planning scan. The accuracy of the correlation between the external marker, which is assumed to be directly related to the diaphragm motion, and the internal tumour position is a crucial question if the tumour motion is to be tracked indirectly.

The correlation between the motion of the diaphragm and external surrogate have been intensively studied using fluoroscopy, based on the assumption of a direct correlation between the tumour and motion of the abdominal surrogate.^21^ However, there is a paucity of data on the correlation between the external surrogate and the internal motion defined using 4DCT for a large cohort of patients. Willmann et al^22^ investigated the internal/external correlation using a fiducial marker and external surrogate in 4DCT and found that fiducial markers provided better internal motion prediction compared to the external surrogate method.

The present study was conceived to examine respiratory-induced lung tumour motion and its correlation with abdominal movement as monitored by the external marker. To this end, a novel method to analyse the RPM external marker signal was developed and applied to test the correlation of tumour motion across a range of patient and tumour characteristics.

## Methods and materials

363 lung cancer patients who underwent SABR treatment between July 2013 and September 2020 at the Northern Ireland Cancer Centre (NICC) were selected for this retrospective study. The candidates underwent free-breathing (FB) 4DCT as a part of their treatment with no additional selection criteria. Patients with multiple masses contoured as a single structure were excluded from this study. The use of the data within this study population was approved by the Belfast Health and Social Care Trust (BHSCT) with research governance permission number 19067CMcG-SS, REC Ref 20/HSC/0002.

### Four-dimensional computed tomography

Free-breathing 4DCT data sets were generated using the GE large-bore Optima 580 and Discovery 590 RT (GE Healthcare, Waukesha, WI) machines. The scanners were coupled with the Varian RPM v. 1.6 (Varian Medical Systems, Palo Alto, CA) for monitoring patient breathing. The RPM system uses an infrared tracking camera that captures the reflected signal in the anteroposterior (AP) direction from a lightweight marker block placed on the patient’s abdomen. The CT reconstruction software retrospectively uses the RPM signal to rearrange the breathing sequence of the acquired volumetric data.

Scanners acquired 4DCT with the cine acquisition protocol for all patients. With the aid of the Advantage 4D workstation (GE Healthcare, Waukesha, WI), the phase binning approach was applied to bin the slices into 10 breathing phases, with the 0 and 50% phases corresponding to the end-inhalation and end-exhalation phases, respectively. Patients were imaged with a 4DCT protocol of 120 KV, 250 mA, 0.5 s tube rotation time, and slice thickness of 2.5 mm. The 4DCT data sets were imported into the Varian Eclipse treatment planning system (TPS) v. 16.01 (Varian Medical Systems, Palo Alto, CA). The tumour segmentations, were completed on the phases 0% (end-inhalation), 50% (end-exhalation), and the maximum intensity projection image (GTV-MIP). From these, a composite ITV structure was generated, reflecting a motion-compensated GTV. Dose prescriptions were made to the ITV plus a further 5 mm margin, *i.e*. the planning target volume.

### Respiratory motion analysis

Tumours were classified, based on their location in the pulmonary zone, into upper, middle and lower, by dividing the lung into three regions of approximately equal height in the sagittal plane. The tumours were also classified based on the anatomical lung lobes, by direct visualisation of the lung fissures. ie. right upper, right middle, right lower lobes and the left upper and lower lobes. No left and right lung tumour grouping was made, as it was previously shown that there is no significant difference in the mean motion amplitude between left and right lungs.^23^ Tumour classification based on the attachment to rigid structures was performed by a clinical oncologist. Given that tumour motion is secondary to the unrestricted inflation of lung, tumours deemed to be possibly invading into the chest wall were defined as ‘attached’, and labelled ‘free’ if not, so that restricted motion could be accounted for.

#### Determination of tumour internal motion

The coordinates of the centre of mass at phases 0% (GTV0%) and 50% (GTV50%) were recorded (Figure 1.A). The composite motion of the centre of mass (3D_0%-50%_ motion) was calculated using equation 1, along with the motion in the superior-inferior (SI), AP, and left-right (LR) directions (Figure 1.B).

(Eq 1) $$3D\;Motion\;amplitude=\sqrt{{\left(X1-X2\right)}^{2}+{\left(Y1-Y2\right)}^{2}+{\left(Z1-Z2\right)}^{2}}$$

In this equation, (X1, Y1, Z1) and (X2, Y2, Z2) are the coordinates of the centre of the tumour in phase 0% and phase 50%, respectively. Figure 1.C shows the end-inhalation and end-exhalation phases for a typical sine wave breathing cycle.

A patient’s breathing characteristics can influence the position of the end-inhalation and end-exhalation. Indeed, phase 0% is not always the phase of maximum inhalation, and similarly, phase 50% is not always the phase of maximum exhalation for some patients. To account for this variation, the full extent of the tumour motion was accounted for by introducing an ITV type motion approach integrating the GTV-MIP with the GTV0% and GTV50% to generate the ITV structure. In some cases, the ITV is reviewed against the GTV from all phases to make sure it encompasses the whole tumour extent.

**Figure 2.**
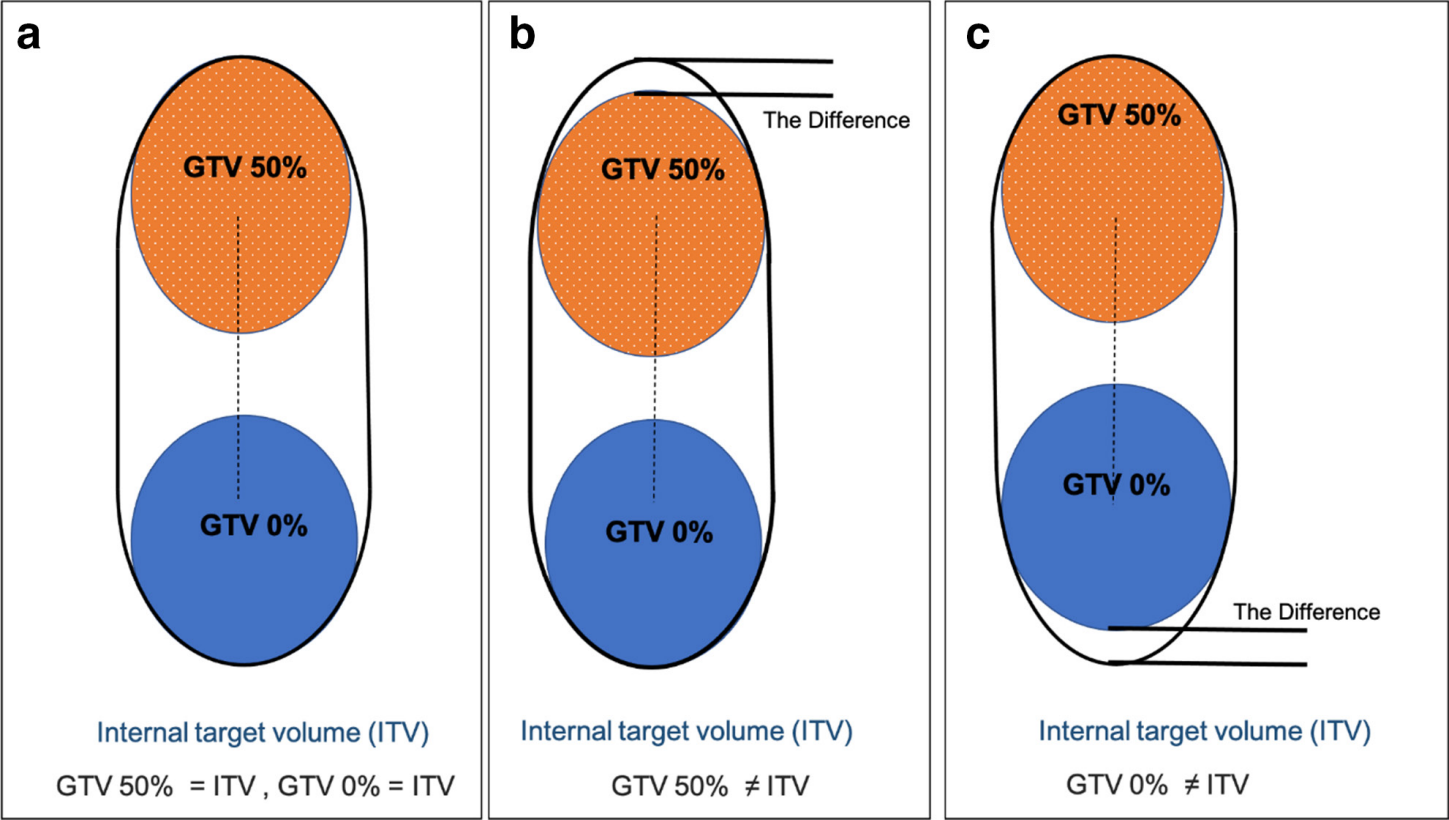
Possible scenarios of the end-inhalation (GTV0%) and end-exhalation (GTV50%) tumour volumes with the ITV structure. GTV, gross tumour volume; ITV, internal target volume.

Figure 2 shows three example scenarios of the end-inhalation and the end-exhalation tumour volumes with the ITV structure. Panel A represents the case where phases 0 and 50% are the phases where the actual end-inhale and end-exhale occurred. In this case, the ITV structure encompasses the GTVs fully in that the most superior slice of GTV 50% and the the most inferior slice of GTV 0% coincide with the respective ITV boundaries. Panels B and C illustrate the cases where there is a difference between the extent of the ITV structure and the two GTVs. In such cases, phases 0 and 50% are not the phases where the actual end-exhale and end-inhale occurred. The discrepancies between GTV0% and the GTV50% with the ITV structure (illustrated between the black lines) which result from the inclusion of the GTV-MIP were added to the centre of mass (3D_0%-50%_ motion) and SI measurements to account for the maximum tumour motion in this study. The latter recalculated motion is referred to, in this work, as the ‘3D maximum motion (3D_m_), and the SI maximum motion (SI_m_).

#### RPM breathing signal (External)

The external respiratory motion analysis was based on the RPM external marker. This allows the analysis of a large number of breathing signals which also require no further manual processing. Out of the 363 patients, 260 patient breathing files were accessible for the analysis. Each breathing trace (RPM signal) was analysed by means of a customised MATLAB (MathWorks InC., Natick, MA) script to define the external breathing motion (median amplitude in the AP direction) which could then be correlated with the internal motion measured from the 4DCT.

**Figure 3.**
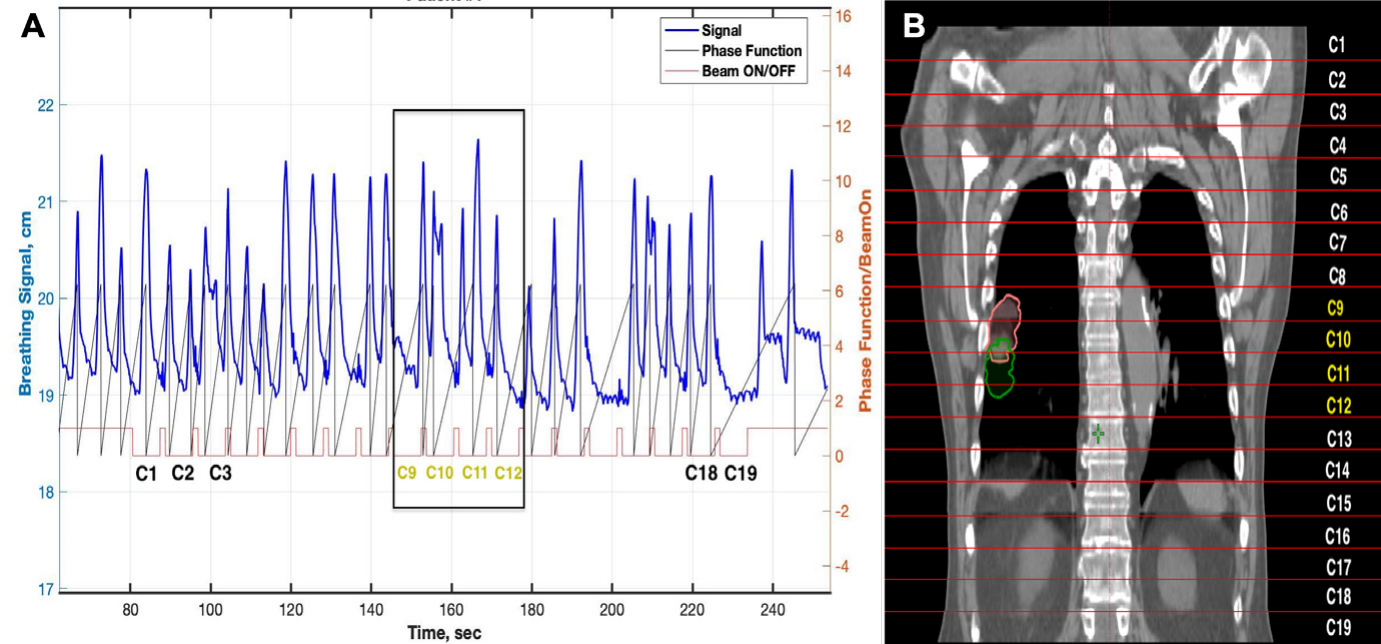
Illustration of a patient breathing signal from the RPM with couch positions numbered (C1, C2, ... etc). Panel B illustrates the average intensity projection CT set with all couch positions numbered. The breathing signal corresponding to each couch position is presented in panel A. The yellow labels in B show the couch positions at which the tumour is being captured (as indicated on a). RPM, Real Time Position Management.

A summary of the key elements of the script are summarised. The patients’ breathing periods and amplitudes were measured from the RPM signal. For the amplitude measurements, the breathing signals from the beginning of the X-ray irradiation (first BeamOn) until the last BeamOff were considered as illustrated in Figure 3 (C1–C19). Further, the script was applied to define the area where the tumour was sampled during the image acquisition to correlate the internal tumour motion with the external surrogate. This was achieved by retrieving the axial scan length, tumour first and last slice, the number of slices/rotations and the slice thickness from the segmented 4DCT.

### Internal–external correlation

The correlation between the internal tumour motion, acquired from 4DCT, and the external surrogate motion was investigated by considering the entire X-ray BeamOn signal as illustrated in Figure 3. It was also assessed against the surrogate median amplitude while the tumour was being sampled as illustrated in Figure 3 (C9, C10, C11, and C12). The external motion from the X-ray BeamOn signal, and separately, the RPM signal at the tumour sampling area was correlated with the internal 3D, SI, 3D_m_, and the SI_m_ motion via Spearman’s correlation for each anatomical lobe. The correlation using the entire RPM signal from the beginning (C1) to the end of the scan (C19) is referred to as the ‘RPM signal’. The correlation with the section of the signal corresponding to the tumour sampling area C9 to C12 is referred to as the ‘Tumour RPM’. The relationship between the tumour volume, analysed in the exhalation phase, and the magnitude of motion (3D_0%-50%_ and SI) was also evaluated.

The relative position of the analysed tumours within the lung was acquired using the average intensity projection (AIP) 4DCT data set. The GTV50% relative position in the SI direction was measured as the ratio of the distance from the apex of the lung to the centre of the GTV structure and the distance from the apex of the lung to the diaphragm passing through the GTV. These distances were measured using the frontal view of the AIP data set. The relative position in the AP direction was the ratio of the distance from the posterior lung boundary to the centre of the GTV and the total distance in the AP direction from the posterior lung boundary. The LR relative position is the ratio of the distance from the centre of the vertebra to the centre of the GTV and the total distance in the LR direction from the centre of the vertebra to the distal edge of the lung contour. The relative positions in the AP and LR directions were measured using the transverse plane.

Statistical analysis was performed using SPSS v. 27 (SPSS Inc., Chicago, IL). The Spearman’s correlation was used to assess the internal/external correlation, and the Pearson correlation was used to assess the linearity between the tumour volume and motion. Correlation between the motion of the centre of mass in the 3D, SI, AP, and LR directions across lobes was examined by Kruskal–Wallis with Bonferroni adjustment and Mann–Whitney tests (significance set at *p* < 0.05). The null hypothesis was set such that the distribution of motion is the same across all groups.

## Results

### Tumour internal motion

A total of 363 tumours were analysed and found to have a median GTV0% volume of 5.3 cc (range: 0.2–66.2 cc), median GTV50% of 5.3 cc (range: 0.2–65.5 cc), and median ITV of 8.5 cc (range: 0.6–142.7 cc). More than half (61.7%) of the analysed tumours were located in the upper lobe of the lung, 6.6% in the middle lobe and 31.7% in the lower lobe. The normality of the distribution of the 3D_0%-50%_ motion was tested by the Shapiro–Wilk test. As the data were found not to be normally distributed (*p* < 0.001), the median and the range are reported throughout the paper.

Table 1 shows the median tumour excursion (3D_0%-50%_ motion amplitude) was 4.2 mm (range: 0.1–26.7 mm) across all tumours. 16.5% of the analysed tumours had a motion greater than 10 mm, only 3% had a motion amplitude greater than or equal to 20 mm. The median amplitude was 3.0 mm (range: 0–26.5 mm), 1.5 mm (range: 0–13.4 mm), and 1.0 mm (range: 0–8.1 mm) in the SI, AP, and LR directions, respectively. The Kruskal–Wallis test indicated that there is a significant difference in the 3D_0%-50%_ motion across the three categories of anatomical lobes (*p* < 0.001) but there is no difference in the 3D_0%-50%_ motion between the upper and middle lobe (*p* = 0.79).

**Table 1.** Summary of GTV centre of mass motion (in mm) acquired from 4DCT classified by anatomical lobe

	3D Motion				SI				AP				LR			
	Median	Minimum	Maximum	*p*	Median	Minimum	Maximum	*p*	Median	Minimum	Maximum	*p*	Median	Minimum	Maximum	*p*
All positions	4.2	0.0	26.7	<0.001	3.0	0.0	26.5	<0.001	1.5	0.0	13.4	0.53	1.0	0.0	8.1	0.12
Upper lobe *N* = 224	3.4	0.1	20.3		1.7	0.0	15.1		1.4	0.0	13.4		0.9	0.0	6.8	
Middle lobe *N* = 24	4.0	0.5	19.8		3.1	0.0	19.0		1.1	0.0	5.7		1.3	0.0	8.1	
Lower lobe *N* = 115	9.9	0.2	26.7		9.7	0.2	26.5		1.7	0.0	9.8		1.0	0.0	5.4	

The Kruskal–Wallis *p*-value is presented for the three anatomical lobes.

3D, three-dimensional; 4DCT, four-dimensional CT; AP, anteroposterior; GTV, gross tumour volume; LR, left-right; SI, superior-inferior.

In Table 1, the predominant motion direction for 64% of the tumours was the SI direction, with lower lobe tumours most likely to have maximum motion in the SI direction (94%). The percentage of tumours that moved more than 5.0 mm in the SI, AP, LR directions was 32.5%, 7.7%, and 1.7%, respectively. The median tumour excursion (3D_0%-50%_) in the upper, middle and lower lobes was 3.4 mm, 4.0 mm, and 9.9 mm as presented in Table 1. There is a significant difference in the SI motion across all lobes (*p* < 0.001), but not between the upper and middle lobe, *p* = 0.24. However, there is no significant difference in the AP and LR motion across anatomical lobes (*p* = 0.53) and (*p* = 0.12), respectively.

**Figure 4.**
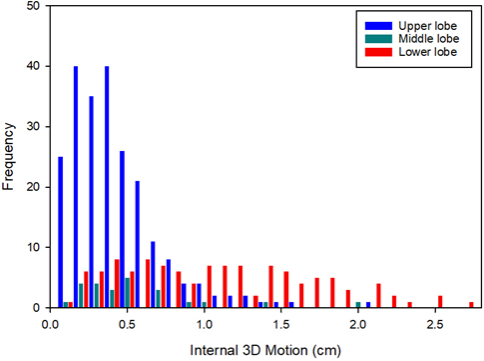
Histogram showing the 3D tumour motion measured in the 4DCT for upper, middle and lower anatomical lobes. 3D, three-dimensional; 4DCT, four-dimensional CT.

**Figure 5.**
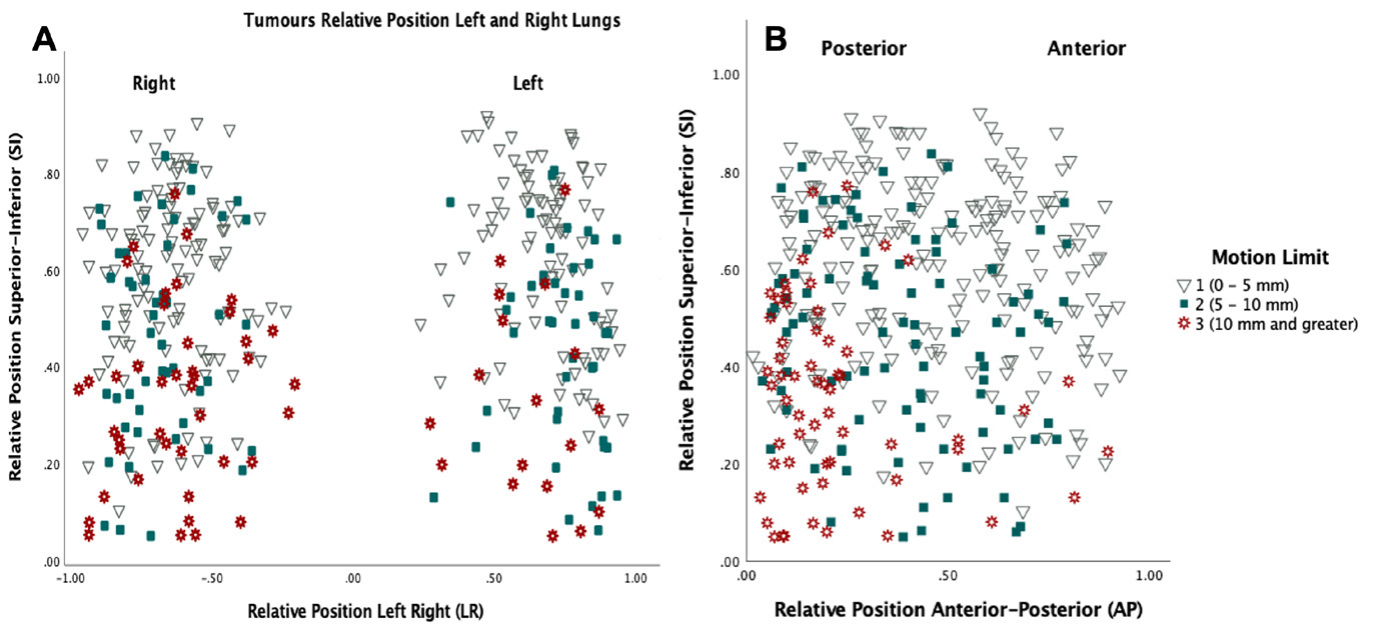
Tumour relative position based on tumour motion limits left-right (**a**) and anteroposterior (**b**).

**Figure 6.**
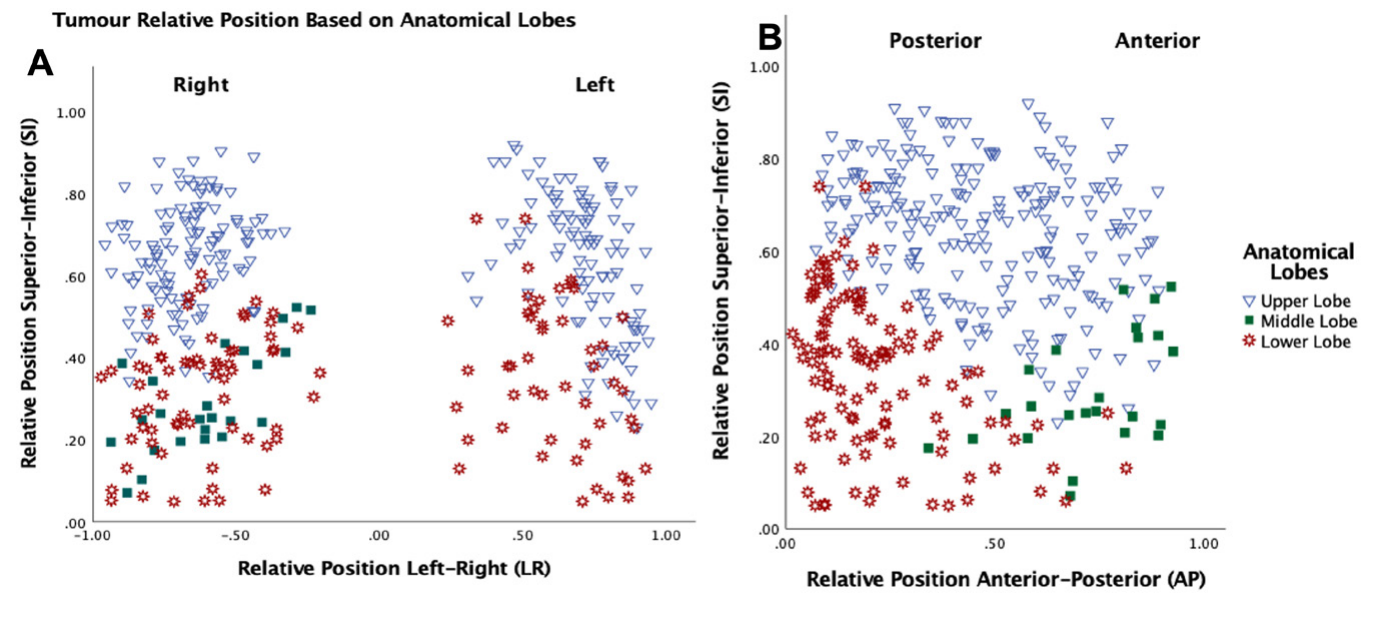
Tumour relative position based on anatomical lobes left-right (**a**) and anteroposterior (**b**).

The histogram in Figure 4 illustrates the motion of the centre of mass based on tumour location. Upper lobe tumours tend to have a smaller amplitude of motion with 96.4% having motion less than 10 mm. Middle lobe tumours had higher amplitudes compared with upper lobe tumours with a maximum excursion of 19.8 mm. The highest tumour motion was found within tumours located in the lower lobe of the lung, with a maximum amplitude of 26.7 mm. 43.5% of the lower lobe tumours have a motion amplitude of more than 10 mm, compared with 8.3% for middle lobe tumours and 3.6% for upper lobe tumours. Figure 5 and Figure 6 show a map of the relative position of the analysed tumours based on both tumour motion and anatomical lobe distribution. The relative tumour position based on the amount of 3D_0%-50%_ motion, was classified into three groups, motion <5 mm, motion ≥5 mm and motion ≥10 mm.

### Internal–external correlation

**Figure 7.**
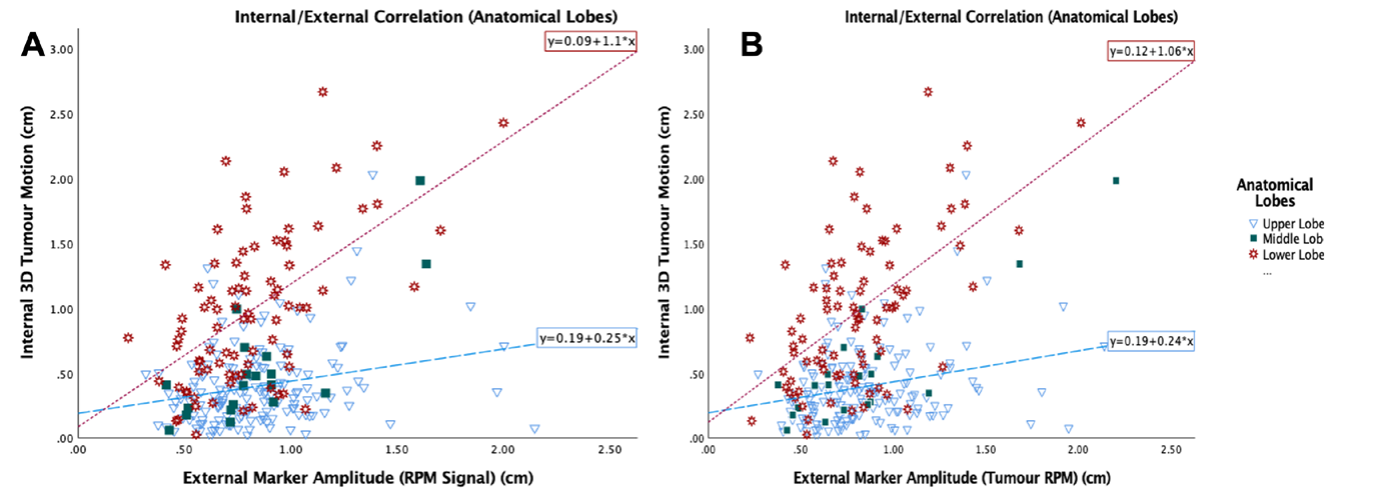
Internal/external correlation from the breathing signal 'RPM signal' (**a**) and the signal corresponding to the tumour sampling area 'Tumour RPM' (**b**) for anatomical lobes. RPM, Real Time Position Management.

The RPM breathing signal is a 1D signal acquired in the AP direction from the top of the patient’s abdomen. The Spearman’s correlation between the external marker amplitude from the ‘RPM signal’ and the amplitude at tumour sampling area ‘Tumour RPM’ showed a strong correlation *r* = 0.95, and the Mann–Whitney analysis indicated no significant difference between the two amplitudes (*p* = 0.92). The Spearman’s correlation between the internal 3D_0%-50%_ motion was weak when all 260 tumours were considered (*r* = 0.24, *p*<0.001). However, differences in the strength of the correlation were apparent for different lung lobes (upper *r* = 0.21, middle *r* = 0.51, and lower *r* = 0.52), as illustrated in Figure 7. The correlation of the maximum motion is presented in Table 2 .

**Table 2.** Spearman’s correlation coefficient of the internal tumour 3D maximum motion (3D_m_), and the SI maximum motion (SI_m_), and the motion of the external marker block from all x-ray BeamOn signal ‘RPM signal’ and at tumour level ‘Tumour RPM’.

Anatomical lobes	Internal 3 _Dm_ Motion		Internal SI_m_ Motion	
	RPM signal	Tumour RPM	RPM signal	Tumour RPM
Upper lobe (*n* = 159)	*r* = 0.10 *p* = 0.19	*r* = 0.10 *p* = 0.203	*r* = 0.10 p = 0.23	*r* = 0.10 *p* = 0.31
Middle lobe (*n* = 19)	*r* = 0.16 p = 0.55	*r* = 0.10 p = 0.71	*r* = 0.10 p = 0.76	*r* = −0.03 p = 0.90
Lower lobe (*n* = 82)	*r* = 0.53 p<0.001	*r* = 0.52 p<0.001	*r* = 0.52 p<0.001	*r* = 0.52 p<0.001

3D, three-dimensional; RPM, Real Time Position Management.; SI, superior-inferior.

The correlation of the SI motion from the ‘RPM signal’ was weak in the upper lobe (*r* = 0.19), and moderate in the middle (*r* = 0.41), and lower lobe (*r* = 0.56). The correlation, when the breathing trace was restricted to when the tumour was being scanned ‘Tumour RPM’, showed a similar trend with no difference in correlation coefficient. The comparison with the tumour maximum motion 3D_m_ and SI_m_ did show similar correlation coefficients for the upper and lower lobes, but not in the middle lobe (Table 2).

The dependence of the 3D tumour motion on the GTV volume showed no linear relationship (*r* = 0.01, *p* = 0.78). As the motion in the SI direction is dominant, the effect of the tumour volume was examined in the SI direction but no linear relationship was found (*r* = 0.04, *p* = 0.48). The classification of tumours based on the attachment to rigid structures indicated that only three tumours were possibly attached (3D_0%-50%_ motion range: 2.9 mm), and due to this limited number of attached tumours no statistical analysis was performed.

## Discussion

The aim of this study was to assess tumour motion in a large sample of lung SABR patients and to analyse the extent of an external surrogate’s correlation with internal tumour motion. This retrospective study was based on 4DCT tumour data acquired with RPM external surrogate, as this is a widely applied imaging/breathing monitoring protocol for SABR treatments.

A recent study by Willmann et al assessed the internal/external correlation by building a population correlative model using a linear mixed model.^22^ In this small population, the RPM marker block was delineated and the excursion between the end-inhalation and end-exhalation phases (0 and 50%) was determined using 4DCT scans. A total of 24 patients were used to build a correlative model, 11 of which were included in the internal/external assessment. The internal tumour motion in the SI and AP direction was assessed against the AP motion of the external marker block. The results illustrated the best association between the AP motion of the external marker and the SI motion of the GTV (*p* < 0.0001; using the restricted maximum likelihood). This work also studied the correlation between the internal tumour motion and the motion of implanted transponders in the SI, AP and LR directions, and concluded that the transponders better reflected tumour motion prediction than the external marker block.^22^

Another internal tumour motion-based study in a large number of radically treated tumours was published in 2015 by Tan et al.^24^ In their work, 101 radical tumours (tumour volume ranges between less than 10 and more than 100 cc) were used to measure the motion of lung tumours in 4DCT and to investigate the correlation between patient and tumour characteristics (age, gender, tumour volume, and respiratory function) and motion amplitudes. Their results showed that radical tumours located in the lower lobe of the lung had higher motion amplitudes in the SI direction compared with the other lobes (*p* < 0.001). The AP and mediolateral motion amplitudes did not differ between lobes (*p* = 0.45 and *p* = 0.32, respectively). In addition, the amount and direction of the motion of tumours was not predictable by their individual characteristics. The research presented in this manuscript confirms these findings in a larger cohort, albeit with tumours smaller in size.

In this work, the analysis of 363 SABR tumours showed that tumours in the upper lobe of the lung typically have the least amount of motion compared with middle and lower lobe tumours. Tumours closer to the diaphragm showed the highest amount of motion amplitude, as expected. Moreover, for the majority of the cases, the tumour motion was predominantly in the SI direction. These findings are in accordance with previously published work.^23–27^ This trend is not always the case however, as upper lobe tumours might have an unexpectedly higher motion amplitude. Hoisak et al, reported the largest motion for a tumour in the upper right lobe of the lung of 27.6 mm in a small cohort of mixed early and locally advanced lung cancers.^28^ Our findings demonstrate that tumour location in the vicinity of the diaphragm is not necessarily a good predictor of increased motion as we also report an upper lobe tumour with motion amplitude of 20.3 mm (Table 1). This tumour was located in the superior-posterior part of the lung and these tumours have been found to have the highest motion after the lower lobe tumours.^29^ Despite the general trend of tumour motion (low in the upper lobe, high in the lower lobe), Figure 5 indicates that lower lobe tumours and tumours in the posterior part of the lung tend to have increased amount of motion.

A comparison between the amount of centre of mass tumour motion of this study and selected previously published studies^23,24,30,31^ is presented in Supplementary Table 1 in the supplementary document. The tumour motion in the SI direction is statistically significant across anatomical lobes (*p* < 0.001). Similar *p* values were presented for the tumour motion in the AP and LR directions. Higher mean and median tumour motion in the SI, AP, and LR were reported by Knybel et al,^23^ and Liang et al,^31^ where the measurements were based on the motion of real-time tumour data.

Supplementary Table 1.

The accuracy of gated and tracked radiotherapy treatments that can be based on the use of an external surrogate can be affected by the degree of correlation between the movement of the external marker and the movement of the patient’s internal anatomy. In such a scenario, it is important to ensure there is spatial and temporal correlation between the moving tumour and the external surrogate for an adequate period of time. In the present analysis, the correlation was assessed between the median amplitude measured from the RPM external marker signal and the internal 3D tumour motion. Willmann et al compared the tumour motion in the SI and AP directions with the motion of the external reflecting marker block structure imaged during the 4DCT (motion between the phases 0 and 50%) using a linear mixed model. The best association was found between the AP motion of the external marker structure and SI motion of the GTV (*p* < 0.0001), which is comparable to the data presented in this study.^22^

To consider the maximum tumour motion extent in all directions, the ITV motion approach was introduced. 4DCT-based tumour motion quantification studies have considered both the motion of the centre of mass^24,25,29–32^ and the maximum tumour motion.^25,33^ The proposed methodology for the whole tumour motion in this work is slightly different than that presented by Antony et al^33^ although the extent of motion was not explicitly presented.

Our analysis included the measurements of tumour motion using the tumour centroid motion (3D_0%-50%_, SI) and the maximum motion using the ITV motion approach (3D_m_, SI_m_). There was no difference in the correlations obtained with these different approaches.

Generally, when the external breathing movement was only considered for couch positions at which the tumour was being sampled, using the ‘Tumour RPM’ as opposed to the full duration of the scan, stronger correlations were not found (Table 2). Although it would conceivably be superior to analyse only the breathing cycles measured at the time when the tumour is being scanned, fewer cycles are sampled and some of these will be incomplete. This is particularly problematic for patients with irregular breathing patterns. For example, even though Figure 3A shows a relatively regular breathing pattern, the tumour sampling period still only encompasses five breathing periods. So, by analysing the breathing motion over a more extended period, a more representative estimate of the mean and median amplitude is obtained for correlation testing.

The association between tumour volume and the amount of motion amplitude has been previously studied extensively for non-SABR targets. Stevens^34^ found no correlation between respiratory-induced motion, tumour location and tumour size using orthogonal radiographs to measure the motion. Recently, with the utility of 4DCT, this study and several other studies have also concluded there is no relationship between tumour motion and volume.^23,29,35,36^ Of note, a contradictory result was reported by one group of investigators using 166 non-SABR targets for 4DCT tumour motion analysis, where the motion was found to be dependent on T-stage, and tumour volume.^32^ Other studies have reported that smaller tumours move more than larger tumours using 4DCT and alternate imaging modalities such as, dynamic MRI.^27,32^ This trend was also reported by Tan et al albeit without statistical significance.^24^ The findings of the present study, along with previously published work, suggest the absence of a strong association between tumour location and volume to the motion amplitude.

The effect of tumour attachments to rigid structures in non-SABR targets have shown a trend in some studies, but this was found to be non-linear. An early study by Seppenwoolde et al has demonstrated that free lower lobe tumours tend to have more motion compared with attached tumours or tumours in the upper lobe.^37^ Wang et al showed that attached tumours tend to have restricted amount of motion. They also reported a non-linear relationship of the tumour motion with respect to the tumour volume (*p* = 0.046) and the attachment degree to a rigid structure (*p* = 0.008) (*n* = 44).^29^ Similarly, Wu et al reported lower motion amplitudes for tumours attached to fixed structures and tumours with large volumes (*n* = 42).^38^ As SABR is considered only for early-stage targets (T1N0 and T2N0), the vast majority of the targets considered in this study were not invasive in rigid structures (*n* = 360), which mitigates the need for tumour attachment analysis.

One of the limitations of this work is the use of breathing files generated by the RPM signals during the 4DCT acquisition, which might be relatively short compared with the nominal SABR treatment delivery time. Although the RPM signals are for treatment planning purposes, significant deviation from intrafraction motion may or may not occur. In comparison with other studies that considered different means of tumour motion quantification and internal/external correlation, this work can give an insight into the behaviour of SABR targets during irradiation prospectively. The results presented in this work can aid future auto-segmentation tools and enlighten other research areas where motion management is in its infancy, such as MRI planning for early-stage lung cancer.

## Conclusion

This study provides a retrospective analysis of tumour motion in lung SABR patients. To our knowledge, this is the largest sample of SABR patient data analysed for both tumour respiratory-induced motion and internal/external correlation using the widely accessible RPM external surrogate. Our findings are consistent with previous publications but the present patient sample was at least two and a half times greater than previous reports. In addition, this study has analysed how internal motion correlates with the motion of an external marker and has shown that there is a good correlation between the two when the tumour is located in the lower part of the lung.
