# Supplementary material for: Evaluation of tumour motion and internal/external correlation in lung SABR
Source: Br J Radiol. 2023 Jul 10;96(1149):20220461. doi: 10.1259/bjr.20220461 (PMC10461274; doi:10.1259/bjr.20220461)
Supplement: Supplementary Table 1. [file bjr.20220461.suppl-01.docx]

Table S 1: Summary of selected studies of respiratory induced lung tumour motion.

| **Author** | **Note** | **Number of Patients** | **Motion in SI (Centre of mass)** | **Motion in AP (Centre of mass)** | **Motion in LR (Centre of mass)** |
| --- | --- | --- | --- | --- | --- |
|  |  |  | **(mm)** | **(mm)** | **(mm)** |
| **(Atkins, et al. 2015)** |  | 41  SABR Patients  (43 Tumours) | All tumours | All tumours | All tumours |
|  |  |  | (Ave: 5.1 ± 4.4) | (Ave: 2.6 ± 1.5) | (Ave: 1.6 ± 1.1) |
|  |  |  | Upper Lobe: | Upper Lobe: | Upper Lobe: |
|  |  |  | (Ave: 2.4 ± 1.8) | (Ave: 2.6 ± 1.6) | (Ave: 1.5 ± 1.1) |
|  |  |  | Lower Lobe: | Lower Lobe: | Lower Lobe: |
|  |  |  | (Ave: 10.5 ± 3.1)  p<0.001 | (Ave: 2.2± 0.9)  p=0.7 | (Ave: 1.6± 1.2)  p=0.9 |
| **(Tan, et al. 2015)** |  | 101 | Upper Lobe:(median: 0.9) | Upper Lobe:(median: 1.3) | Upper Lobe:(median: 0.8) |
|  |  | (Radical Patients) | Middle Lobe:(median: 2.6) | Middle Lobe:(median: 1.6) | Middle Lobe:(median: 1.3) |
|  |  |  | Lower Lobe:(median: 7)  p<0.001 | Lower Lobe:(median: 1.6)  p=0.45 | Lower Lobe:(median: 0.8)  p=0.32 |
| **(Knybel, et al. 2016)** |  | 145  SABR | All tumours  (Ave: 6 ± 2.2), (Range 1.85 – 18.75)  Upper Lobe:  (Ave: 3.6 ± 1.6)  Lower Lobe:  (Ave: 8.2 ± 4.2)  p<0.001 | All tumours  (Ave: 2.8 ± 1.6),  (Range 1.01- 8.86) | All tumours  (Ave: 2.2 ± 1.3),  (Range 2.10 - 6.36) |
|  | Measured during treatment |  |  |  |  |
| **(Liang, et al.2019)** | RTT  (Motion from 4DCT presented) | 83 SABR Patients  (86 lesions) | Upper and Middle Lobe  (Ave: 2.7 ± 2.7)  Lower Lobe: (Ave: 9.3 ± 4.1) | Upper and Middle Lobe:  (Ave: 1.3 ± 1.2)  Lower Lobe: (Ave: 1.9 ± 1.4) | Upper and Middle Lobe:  (Ave: 0.9 ± 0.9)  Lower Lobe: (Ave: 1.1 ± 0.9) |
| **Current Study** | Binned 4DCT | 363  SABR | All tumours  (Median: 3.0), (Range: 0.0 – 26.5)  Upper Lobe:  (Median: 1.7), (Range: 0.0 – 15.1)  Middle Lobe:  (Median: 3.1), (Range: 0.0 – 19.0)  Lower Lobe:  (Median: 9.7), (Range: 0.2 – 26.5)  p<0.001 | All tumours  (Median:1.5), (Range: 0.0 – 13.4)  Upper Lobe:  (Median: 1.4), (Range: 0.0 – 13.4)  Middle Lobe:  (Median: 1.1), (Range: 0.0 – 5.7)  Lower Lobe:  (Median: 1.7), (Range: 0.0 – 9.8)  p=0.53 | All tumours  (Median:1.0), (Range: 0.0 – 8.1)  Upper Lobe:  (Median: 0.9), (Range: 0.0 – 6.8)  Middle Lobe:  (Median: 1.3), (Range: 0.0 – 8.1)  Lower Lobe:  (Median: 1.0), (Range: 0.0 – 5.4)  p=0.12 |
|  |  |  |  |  |  |
